# Prevalence and Associated Risk Factors of Toxoplasma gondii IgG Antibodies in Patients Diagnosed with Depression from Western Romania: A Case–Control Study

**DOI:** 10.1192/j.eurpsy.2025.865

**Published:** 2025-08-26

**Authors:** A. G. Mihu, S. Grada, L. E. Piros, S. A. Sprintar, A. M. Lupu, T. R. Olariu

**Affiliations:** 1 Department of Biology and Life Sciences; 2 “Aurel Ardelean” Institute of Life Sciences, “Vasile Goldis” Western University, Arad; 3 Discipline of Parasitology, “Victor Babes” University of Medicine and Pharmacy; 4 Clinical Laboratory, Municipal Clinical Emergency Teaching Hospital, Timisoara, Romania

## Abstract

**Introduction:**

Chronic toxoplasmosis has been reported to cause neuroinflammation in humans, linking it to neuropsychiatric disorders, including depression.

**Objectives:**

To assess the prevalence of *Toxoplasma gondii* IgG antibodies in patients diagnosed with depression from Western Romania and compare the findings with a control group of healthy volunteers from the general population without any psychiatric disorders.

**Methods:**

We included 230 participants, consisting of 115 psychiatric patients diagnosed with depression at the Psychiatric Clinic, County Emergency Hospital of Arad, Western Romania. These patients were matched by age and gender with a healthy control group of 115 volunteers. Clinical evaluations, a brief questionnaire to assess the risk factors, and laboratory tests were conducted, including serological testing for the presence of *Toxoplasma gondii* IgG antibodies.

**Results:**

In both the study group and the control group the mean age was 52.96 ± 10.29 years and 72/115 (62.61 %) were female. A significantly higher seroprevalence of *Toxoplasma gondii* IgG antibodies was demonstrated in patients with depression when compared to healthy volunteers (*p* value <0.001). We noted a higher prevalence of *Toxoplasma gondii* IgG antibodies in depressive patients aged between 50-59 years (*p* value = 0.008) and 60-69 years (*p* value = 0.03) when compared to their counterparts from the control group. Females diagnosed with depression presented a significantly higher seroprevalence of *Toxoplasma gondii* IgG antibodies (*p* value <0.001) when compared to healthy female volunteers. No difference between the two groups was noted when we assessed the participants educational level, contact with the soil, consumption of undercooked meat and contact with cat feces.

**Conclusions:**

The presence of *Toxoplasma gondii* IgG antibodies was significantly higher in depressive patients attending the Psychiatric Clinic in Arad County, Western Romania, when compared to healthy controls. Our findings suggest a potential link between chronic *Toxoplasma gondii* infection and depression.

**Disclosure of Interest:**

None Declared

